# The Utility of DSRC and V2X in Road Safety Applications and Intelligent Parking: Similarities, Differences, and the Future of Vehicular Communication

**DOI:** 10.3390/s21217237

**Published:** 2021-10-30

**Authors:** Eduard Zadobrischi, Mihai Dimian, Mihai Negru

**Affiliations:** 1 Department of Computers, Electronics and Automation, Faculty of Electrical Engineering and Computer Science, “Stefan cel Mare” University, No. 13. Str. Universitatii, 720229 Suceava, Romania; dimian@usm.ro; 2 Department of Computer Science, Technical University of Cluj-Napoca, Gh. Baritiu St. 26-28, 400027 Cluj-Napoca, Romania; mihai.negru@cs.utcluj.ro

**Keywords:** vehicle safety applications, safety driving, infrastructure-to-vehicle communication, dedicated short-range communication, vehicle-to-vehicle communication

## Abstract

As the technological advancement in the automotive field increases and the complexity of vehicle and infrastructure applications is extremely high, new directions and approaches are needed in this field. Supporting and developing vehicular applications dedicated to road safety by analyzing the current behavior of existing networks in various forms is imperative. This paper studies and implements a DSRC-type communications infrastructure that receives a set of controllable and adjustable indicators, which can provide messages to network drivers in a timely manner. The implementation is based on the 802.11p protocol and initially addresses pedestrian infrastructure or pedestrian safety, controlled areas, and perimeters that allow intelligent communications. The design and setting of the communication parameters in the lower layer of the DSRC stack for vehicle applications are part of this work, aspects that are also relevant in the case of autonomous vehicles.

## 1. Introduction

Regarding the development and safety policy of road transport in the European Union, we observe many methods aimed at the efficiency and safety of traffic but also of pedestrians. These strategies also include ways to reduce emissions and rules that are as environmentally friendly as possible to develop an eco-driving and safety-driving style. It also highlights strategies that address emerging technologies capable of developing new directions and opportunities dedicated to traffic safety and its management in conditions of highly congested cities and with an excessive increase in vehicle density compared to the number of inhabitants. According to these new directives, technologies, such as V2X (vehicle-to-everything), VLC (visible-light-communications), or V2V (vehicle-to-vehicle communications), are highlighted, dedicated especially to areas exposed to congestion and prone to road accidents. The ability of vehicles to communicate through and with infrastructure is standardized by introducing wireless (IEEE 802.11p) or (IEEE 802.15.7) communication. The massive development of vehicles has pushed towards the shaping of new functionalities and the incorporation of technologies and infrastructures dedicated to solving the previously mentioned problems. Most vehicles from premium classes benefit from info-traffic or driving assistance systems, pedestrian detection, collision prevention, and many others to avoid a delicate future situation that could endanger several lives. The literature and studies offer many implementations that car manufacturers can see as solid prospects in future prototypes. They give them the opportunity to outline new systems dedicated to vehicles with a solid and truthful basis that can significantly reduce accident rates and traffic congestion, as well as shape an ecological and friendly driving environment, whether we are talking about assisted driving or autonomous vehicles. The last period relaunched ideas related to this direction and new systems and devices have been launched to offer lightness in the driving process. Most manufacturers, such as the VAG Group, Volvo, or Daimler Group, are beginning to use and integrate V2X-DSRC platforms for production vehicles. Manufacturers have the opportunity to use dedicated platforms designed by Cohda Wireless or NXP that are already established by their exposure in the segment of autonomous vehicles. These hardware infrastructures are already in the fifth generation and offer the guarantee of secure and viable communication over long distances in conditions of high mobility on frequencies between 700 MHz and 5.9 GHz in the dual-band. Between 2019 and 2020 alone, more than 400,000 people were seriously injured in road accidents, and for some of them, nothing could be done to save their lives. According to the World Health Organization, road accidents are the leading cause of death worldwide among young people aged 15 to 35 [1,2]. In addition to this negative statement, road transport also has significant material and environmental losses, with long-term effects on human health [3,4,5].

Official estimates of traffic congestion, road accidents, and loss of life create decreases of up to 3% [6] of the gross domestic product (GDP) in Europe [7].

A series of forecasts made by the competent institutions look something like this:

- Transport is an extremely important branch in the development of industries and represents a sphere of CO2 emissions in the European Union and over 40% of the final energy consumption [8];
- Fuel consumption and pollution increase in direct proportion to a high percentage of congestion [9];
- European Union statistics and concerns show increases in closely linked freight and passenger transport activities by 2050 of 82% and 50%, respectively [10,11];
- A recent analysis by the German regional economic research center reported that a driver spends at least 40 h in traffic and congestion during a year [12]. The example was drawn from a developed country with a high-standard infrastructure with alternative traffic solutions and routes [13];
- According to official reports from the Global Burden of Disease (GBD) before the outbreak of the COVID-19 pandemic, they reported that by 2020, road accidents were the second leading cause of loss of human and economic lives worldwide, and 90% of accidents are caused by human error [14].

**Figure 1:**
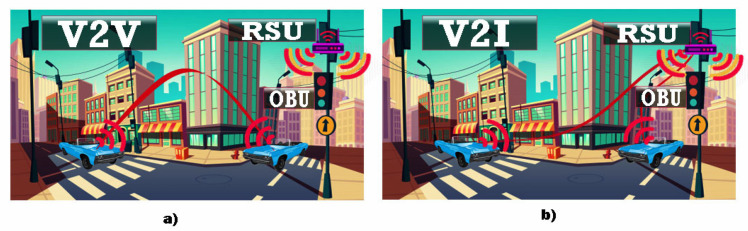
(**a**) Vehicle-to-vehicle communication with RSU-OBU; (**b**) Vehicle-to-infrastructure communication with RSU-OBU.

Many of these alarming statistics have been analyzed intensively and with great care by the scientific community but also by the competent institutions subordinated to transport policy in an attempt to develop viable solutions at the European level [15]. Many of the current problems coagulate in the area of the application of cooperative intelligent transport policies and the integration of dedicated systems (C-ITS) [16]. C-ITS uses V2V or V2I communication, and a representative scenario is illustrated in Figure 1, which shows how to increase traffic safety and reduce the number of accidents through inter-vehicle or between vehicle and infrastructure communication.

Although autonomous or intelligent vehicles can exchange information with each other through cooperative communication systems, we need to investigate major limitations and analyze transfer rates, which should not be delayed by more than 100 milliseconds. Current low-frequency systems have limited perimeters and sometimes delays of the order of 400–500 milliseconds for a coverage radius of about 200m. The results of the current studies are based on complete systems equipped with traffic control units and road units (RSU), and the vehicles are equipped with onboard control units that can become an integral part of the ECU component [17,18]. Most of the current results have analyzed and exposed the systems in different scenarios, which are as complex as possible, but at the same time have not simplified the method of communication. This manuscript addresses the possibility of communicating only based on a simulated ITS system for the data communication part that communicates directly with networked OBUs. This attempts to simplify the process and release the architecture, obtaining high reliability, with extremely relevant distances, without losses, the only aspect sacrificed being the speed of information transmission, with this being supported by the use of small packets of data according to the nomenclature of case codes.

The article comes with a procedure dedicated to communication between vehicles, but also between them and infrastructure based on 5.9 GHz standards in order to obtain distances of over 1000 m. The article highlights the capacity and adaptation of the system even without the use of an RSU, which offers the possibility of extending the communication to other nodes in the network. The benefits of cooperative communications between vehicles and infrastructure substantially improve the road sector by imposing a high degree of full awareness of the dangers. The remainder of this article is organized as follows. Section 2 addresses existing problems and solutions in the field of road safety and vehicle applications, highlighting the challenges and solutions set out in the literature. Section 3 presents the proposed system and the architecture on which it is based, including hardware and software components, and details the software architecture and tests with experimental data of the proposed solutions in the field of road safety. Section 4 provides a discussion that sets out the experimental results obtained that confirm their usefulness and necessity, while Section 5 provides conclusions.

### 1.1. Analysis of Communications Depending on the Standard

We can say that through the communication and harmonization offered by the interoperability of different ITS systems, we outline a basic requirement expected by the applications dedicated to this field, managing a minimum coverage offered by V2X to the existing road infrastructures. This extremely important factor, in which vehicles are equipped with factory-integrated devices, is an evolving factor in terms of intelligent transport. Services dedicated to road and safety communications are urgently needed as the end-user is human, completely unpredictable, and sometimes irresponsible in their choices and reactions. The capacity of these technologies only exposes the profitability and advantages of using equipment with a global and European standardization, while at the same time being extremely mobile. The efforts of the authorities and the European Commission in 2009 succeeded in standardizing M/453 to all three European-level organizations (ESOs: ETSI, CEN1, and CENELEC2. The requests came based on a coherent set of standards, guidelines, and specifications to be able to implement new types of cooperative intelligent transport at the EU level [18]. As for CEN and ETSI, they officially accepted the standard, except CENELEC2 through disinterest and non-attendance at debates and development of the standard. Although the collaboration was wanted at the European level, it benefited from international support with the help of ISO3, IEEE4, and SAE5 for better cooperation and notable results on C-ITS standards [19]. Through the existence of several and working research groups, councils, and technical committees that are directly involved in standardization, divergences emerged, which have sometimes been constructive, leading to qualitative development. We can say that the most important standards dedicated to intelligent transport are:

- ETSI TC ITS;
- CEN/TC 278;
- ISO/TC 204; and
- IEEE 802.11 and 1609 WG.

Following the debates at the ETSI-ITS congers in Berlin (Germany) at the time, ESO approved and confirmed the first official version of the standardization, which was later published successfully [20,21]. Through the development at the standard level and its approval, they managed to offer solutions for car manufacturers, who implemented individualized or extended solutions in a cooperative way.

### 1.2. Development of Communication Protocols—References on Architecture

**Figure 2:**
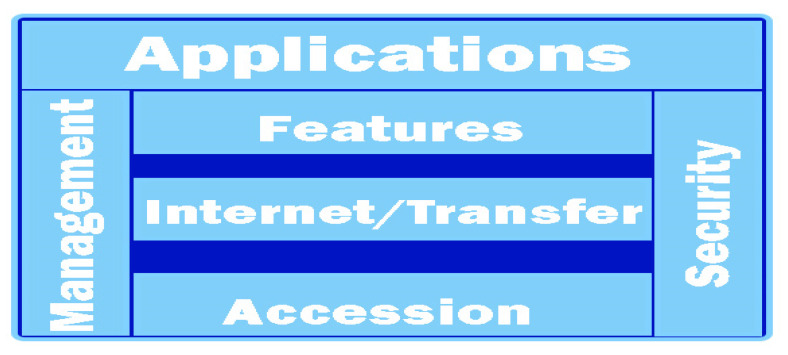
ITS reference architecture—ISO/OSI model.

At the base of the current systems is a reference model of communication for the ITS direction, being defined by the ETSI standard EN 302 665 [22]. We can say that it addresses the type of an open system and allows embedding with other useful architectures in the desired implementations. In a first conclusion, it can be used as a modular tool and can receive further functionalities depending on the architectures of the other systems. Therefore, the ITS reference architecture, represented by Figure 2, is based on the OSI model [23], and is configured as follows:

- Accessibility to OSI layers one and two (thereby maintaining the physical and data connection);
- Communication and transport represented by OSI layers 3 and 4 (communication and transport layer); and
- The benefits are represented by OSI layers five, six, and seven (upper layer, identified by representation and application).

We can say that in the case of the reference architecture, there are three blocks, one of which is dedicated to subsequent applications and developments. The presentation of IT-S applications uses stationary ITS services in several applications based on two or more connections, regardless of whether we are talking about the client or server. In addition to the insulation layers, there are functions that form connections between layers, also called cross layers. According to the two lower levels, the ISO/OSI model presents the physical connection of data with what is identified in the access layers of the ITS reference architecture.

The European standard defined by ETSI EN 302 663 [24] is identified by ITS-G5, which is based on IEEE 802.11 or more precisely IEEE 802.11p (incorporated in IEEE 802.11 in 2012) with a subsequent amendment to 802.11a. The use of orthogonal frequency modulation (OFDM) has a doubled duration (4 vs. 8 μs) and a stagnation period of (0.8 μs vs. 1.6 μs). This factor reduces the interference caused by multipath propagation. Additional enhancements optimize speed reduction (27 vs. 54 Mbps). The high multipath delay results in a low coherence bandwidth, which is attenuated by halving the bandwidth (10 vs. 20 MHz). The initial design of the 802.11p standard was based on the environmental protocol stack (WACE), but it is also used in the European ITS stack (ITS-G5). Insignificant changes in the frequency band exist in the high-end area (5.850–5.925 GHz in the US and 5.855–5.925 GHz in Europe) [25,26] and channel allocation. In the case of a vehicle communication system, many technical challenges are not encountered in the case of wireless networks. The scenarios will not be static, such as a switch or router in an office, but the constant dynamics and mobility of vehicles create a variety of nodes with differentiated traffic between them. We can say that this result is caused by a rapid fading and an extreme Doppler spread [27] causing interference between carriers. All these highlighted deficiencies must be addressed in the design and implementation of the receiver. We can say that ITS systems are usually critical in time, safety, and mix and a failure could cause loss of life, accidents, or injury to other people or participants in trafficking. Unlike cellular environments, applications and vehicles must communicate directly with each other and should not be mediated by a control base or access point (Vehicular AdHoc Network—VANET). The need for authentication becomes a consummator of time and association procedures [28] that increase the latency and thus there is the possibility to delay processes and automatically result in non-fulfillment of road information processes. The 802.11 standard defines the use of access points, including multiple sets of basic services (BSSs) that have a single access point along with all associated connectivity points. In the process of communicating with the vehicle outside the BSS context, the 802.11p standard uses the BSSID7 wildcard, which is an identifier loaded with only one binary in the 802.11p frame antenna. This factor indicates that the receiver does not need a standstill time to complete the authentication and pairing process. The changes allow peer-to-peer communication in the 802.11p standard and automatically reduce the latency significantly. This is a precondition to being able to overcome any inconvenience encountered during the communication. 

### 1.3. Approaching LTE and 802.11p Standards

According to [29], 802.11p is presented as the only wireless technology capable of meeting low latency requirements below 100 ms in the case of information transfer and road safety messages. As for current technology, 4G and 5G or long-term evolution (LTE) can manage and transmit information with a low latency of about 20 ms. LTE can offer high transfer rates reaching at least 100 Mbit/s with downlink connections and 50 Mbit/s split with previous connections but also low latencies [30]. Thus, 3GPP states that the LTE standard can manage very high speeds at the terminals, which provides an ideal perspective and condition in communication between vehicles. It also can adapt to speeds of 350 km/h or even 500 km/h depending on the frequency band, although in these cases, degradation of the performance at high speeds is inevitable. Recent studies have evaluated the applicability of LTE to intelligent transport systems. The University of Twente [31] reported that LTE can meet all ITS requirements in all scenarios, and in some chapters exceeds the 802.11p standard. A comparable feature is the cyclic prefix (CP) LTE, which can support an intense connection with about 700 ITS users compared to 400 ITS users in the case of 802.11p, having a longer coverage range of 2000 m compared to 800 m for 802.11p. In the case of 802.11p, much lower thresholds, which is also the most important property in the case of road applications, characterize the latencies. In the case of transmission overload and connectivity, LTE cannot consistently support strict ITS requirements. The study [32] analyzed and mentioned that LTE can support road safety applications that are weak in terms of cooperation. The network becomes slightly overloaded and tends not to deliver the expected results. The comparisons in [33] show the UMTS cellular system with LTE and 802.11p, but only expose the setup in a single scenario. UMTS cannot meet the requirements of C-ITS, and LTE has provided a perspective, but it does not consistently meet the same latency as 802.11p communications. In a first conclusion, we emphasize that 802.11p cannot be replaced by LTE nor can it be outclassed in terms of implementation and costs. Limiting LTE coverage backlogs is again another negative factor according to a European Commission report.

## 2. Materials and Methods

The main feature of C-ITS services is that when an individual road user interacts with the road infrastructure in relation to their position, the dynamics of objects or routes, state, or degree of mobility, they are found in the form of cooperative awareness (CA). This feature is necessary for ensuring the correct and independent functionality in terms of ITS services and applications, knowing the exact position of the cars or the perimeter in which they run, and detecting the risks of collision. Therefore, a regular exchange of information and features between road users through direct line point-to-point communication provides an increased rate of cooperative awareness messages. 

**Figure 3:**
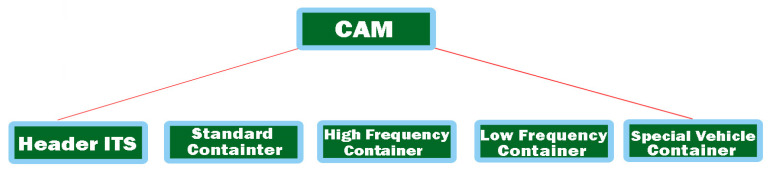
General structure of CAM messages.

The services and functions offered are urgently needed in terms of ITS with the existing infrastructure compared to the messages or services of other communications, which will not be redirected to its network. According to the previous discussions, the sending and receiving of CAMs are done through the basic service offered by CA, with the property and characteristic of the facilities layer, according to the structure in Figure 3. Its role is to manage the frequency of generating CAMs, but also the interval time between two consecutively generated CAMs. Therefore, its lower and upper limit is defined by the ETSI EN 302 637-2 [34] standard at 1 and 10Hz, respectively.

We can say that within these limits, the basic services offered by CA can control the generation frequencies but depend on the degree of change of its own layer, for example, the process of changing position and speed, but especially channel mobility, determined by the control decentralized congestion system (DCC). These generation frequencies are necessary to be able to generate a required CAM response that is below 50 ms to ensure a correct interpretation of the message regardless of the area in which it was transmitted by the ITS stations. We conclude by saying that a timestamp is needed, which can therefore provide synchronization promptly regardless of the road infrastructure users, with this being an extremely crucial aspect in this whole process. When a cooperative awareness message has a certain structure, as shown in Figure 3, the process of failure and synchronization is achievable without improbability. Such a message consists of the ITS packet data unit (PDU) header, and it also includes the protocol version, the message type (CAM, DENM, DCC), and the initiator identification ID and other information stacks. They can be used optionally but also individually; through the ITS station, the vehicle identifies several stacks and containers with high-frequency information. Thus, the main container has the capacity and crucial information about the ITS station, location, time, geographical position, and coordinates on the common nodes in the vicinity for all types of ITS stations (ITS-S vehicle, ITS-S side, or personal, direct, or indirectly). The container that operates at high frequency contains information from the dynamic environment and the one that assimilates the rapid changes of the ITS station, speed, direction, and changes has the lanes.

The container that operates at a low frequency usually assimilates statically and is not very mobile, with dynamic information that comes from the ITS transmitting station, such as headlight brightness, car dimensions, and ground positioning. A third container has information with the specifics offered by the vehicle in traffic, intended entirely for vehicles in urgent traffic and with extremely dangerous goods. In the current standard EN 302 637-2 [35], only TS stations managed by vehicles are accepted, without the high-frequency components being mandatory and the low-frequency one for special regime vehicles being optional. The change of the communication mode via CAM is activated in the conditions of a delay of more than 500 ms from the last transmissible pulse. For the types of messages stated above, the standard models of the CAM are formatted into an Abstract Syntax Notation One (ASN.1) ideal for encoding and decoding CAMs. These methods encode non-linear PER packets, which are extensively presented in [36].

### 2.1. Contribution of Decentralized Environment Notification Messages (DENMs)

**Figure 4:**
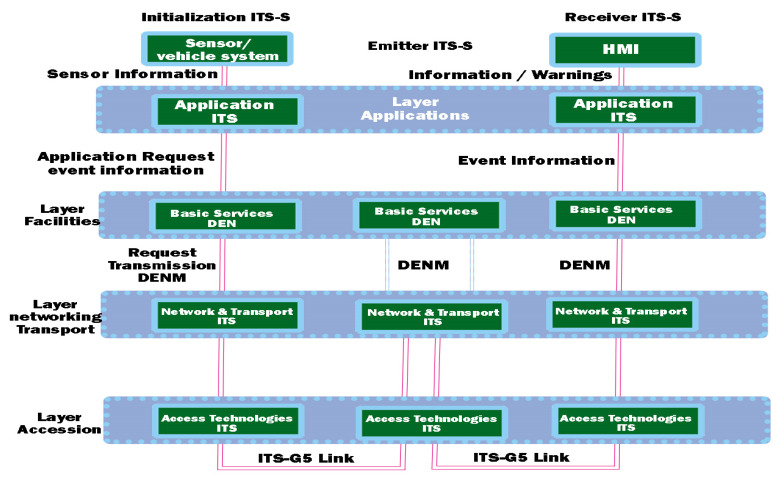
General DENM flow diagram.

We can say that in the case of events that happen instantly on a road, the user needs quick and immediate information, such as a decentralized notification of the triggering environment of an event. This type of DENM protocol is based on a standardized implementation on the DEN service in the facilities layer, containing information about the situation that led to that event and the type of danger, its position, or duration required for detection (according to ETSI 302 637-3 [37]). Outlining an ITS control area can initiate the transmission of a new DNM when an event occurs at the same time, initiating event information and closing previous events with a DENM-type override. Reception, in the case of an ITS station, can also process the DENM analysis at the input of each data layer, analyzing the data flow and exchange according to the representation in Figure 4. The most significant difference between CAM and DENM broadcasting takes into account the branched transmission. Therefore, DENMs can extend the transmission to other road users as well. The highlighted feature ensures that the vehicles are at a considerable distance, much longer than the interval initially established for communication. According to the architecture, the redirection is performed in the ITS internet and transport layer. The ITS central station does not resort to recursion of the DENM message when the range is longer than the coverage, and then the basic DEN service intervenes and redirects the court to DENM. The interaction with other entities of the layer offered by the facilities feature is the one related to the local dynamic mapping (LDM), which contains information for the analyzed areas and provides the attributes of the vehicles in that area within the basic DEN service [38].

By assigning a new ID to initialize different events, you have to go through the ITS station filter and highlight the new attribute of the action individually. The ITS station ID generates a unique code that is subsequently incremented at each iteration within the original ITS-S event. Each container is identified and defined with the duration in which a message is valid and viable through DENM (ValidityDuration), which must include the position or area of the event.

In terms of functionality, such as the state of the container, it aims to define the type of event, generate the unique ID for the eventType event, define and identify the danger, and if necessary divide it into two elements, such as causeCode and subCauseCode. Such an example can be the failure of traffic congestion and it can be identified by the sequence causeCode = 1 or subCauseCode = 0. The existence of a complete table can be analyzed in [39]. When we talk about the location of the container, it aims to store information on how to display the location, the coordinates, being written in the form of a data set with consecutive positions that automatically identify the position of the event. Multiple instantiations provide different information from the initial exposure and compare the events provided by the other nodes in the transmission that compare DENMs to up to 40 PathPont incidence positions.

**Figure 5:**
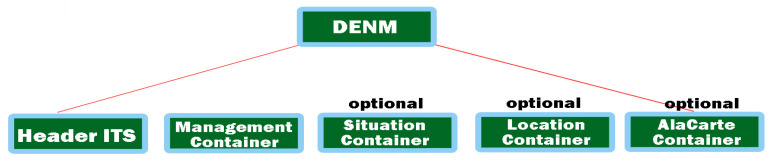
General structure of DENM messages.

The ITS PDU header and the management container are included in the DENM, completing the entire structure, with the others being optional. The container analyzing the location and the event must be permanently synchronized for the validity of the information, except when the event is canceled or DENM denial occurs, being excluded from both functions, as shown in Figure 5.

### 2.2. Cooperative Communication Elements in the Vehicle

**Figure 6:**
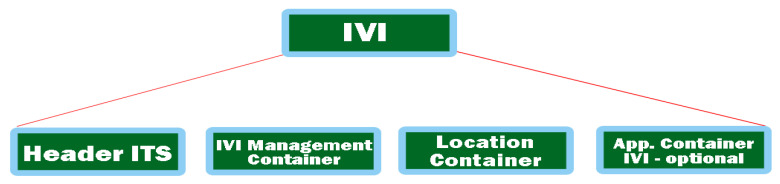
General structure of IVI messages.

A promising objective in this direction is to try to analyze and perceive traffic signals or other elements related to road infrastructure. This information inside the vehicle is necessary because the driver can be informed visually and acoustically at any time. The common problem between the perception of road signs and markings with other travel information is also found in the case of these ITS-type applications having the obligation to incorporate a permissive road nomenclature. This combination of real environmental information with that stored in containers is used to inform the driver in advance about certain events, strictly only those that are really important and of notable relevance. In this direction, each road operator must implement the technologies presented because the number of road signs or markings can be constantly adjusted and even temporary speed limits may be imminent depending on the rehabilitation works of the roads. Therefore, the structure of a general IVI message is represented in Figure 6 and is defined by CEN/ISO TS 19321 [40]. It is composed of several mandatory IVI management stacks and iterates the location containers according to the essential information processing them into relevant messages.

## 3. Experimental Evaluation Simulation and Results

Thus, 5.9 GHz short-distance communication (DSRC) is based on the IEEE 802.11p standard, which has become a very promising solution, and is implemented in vehicle-to-vehicle (V2V), vehicle-to-road (V2R), and vehicle-to-infrastructure (V2I) applications, as well as in the development of autonomous vehicles. DSRC uses orthogonal frequency division multiplexing (OFDM), sometimes based on the wireless local area network (WLAN) standard known as the IEEE 802.11a standard. In a direct comparison with IEEE 802.11a, the DSRC has robust interference sturdiness and can adapt to continuous and fast transition conditions. The structuring of the DSRC channel provides seven channels with a frequency of 10 MHz and these are in turn divided into another 52 sub-channels. To comply with data security requirements, these sub-channels are classified into four priority categories, depending on how they are applied and transmitted to the central channel [41,42].

**Figure 7:**
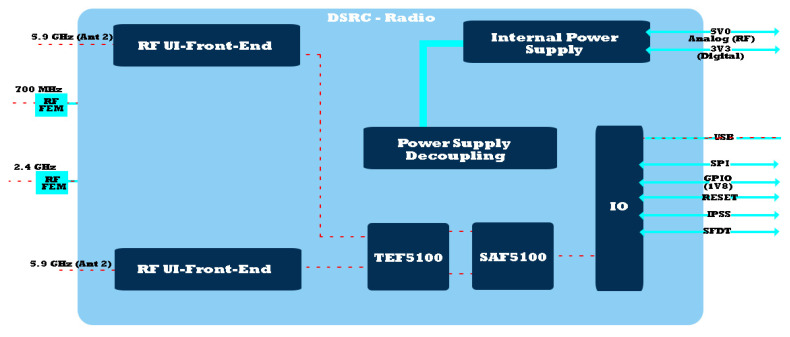
Mk5-OBU module architecture.

Therefore, in the context of data collision prevention, DSRC relies on multiple carrier access in collision detection (CSMA/CA). Being a type of wireless communication with short to medium range, it folds extremely well on the concepts of data collision systems for road safety. The final approach analyzes the synchronization and allocation of slots dedicated to the distribution in multihop networks, with increased mobility conditions, and correctly optimizes IEEE 802.11MAC communication in the approach of systems dedicated to the road sector. Although there are performance limitations in establishing the MAC protocol, in the case of IEEE 802.11, one can speak of self-competition between adjacent nodes and the flow of information, which subsequently leads to a limitation that directs the information to the ad hoc distance vector (AODV). This is most useful in message protection within DSRC applications [43,44]. Figure 7 shows the block diagram of the DSRC module, with a return loss on all RF ports not exceeding −0 dB. The measurements made for the DSRC receiver are in the range of −95 to −20 dBm, with a value of ±2 dB under extreme operating conditions. The versatility and mobility of PHY, based on a complementary IEEE 802.11p physical layer radio transceiver (PHY), processes all data through the modular receiver through advanced algorithms. The PHY–RF user interface can provide the ability to improve radio configurations. This feature allows the module to implement single-channel or dual-radio DSRC systems. The RF subsystem has the possibility of transmission through antenna-type ports built separately for 5.9 GHz frequency bands with an inclined finish on the ends of the module to cover a wide area [45,46]. RF outputs are plotted through separate Rx and Tx pins (receiver and transmitter) capable of communication in the case of the 760 MHz and 2.4 GHz frequency bands. To use them, front-end RF circuits and off-board UIs are needed to be integrated into a radio system, as shown in Table 1.

We can say that in the dual-radio configuration, the module benefits from an extreme PHY mobility that behaves like two independent PHY modules, with each operating on a different radio channel. The U-Blox reception is sensitive to single and dual reception antennas operating at 5.9 GHz in the 10 MHz bandwidth DSRC mode. The packet error rate (PER) is less than 10% at a PSDU length of 1000 bytes for these input levels. The reception sensitivity is measured with an input signal directly to the antenna ports at the external temperature (±6°). Each touch is dimmed using pure Doppler, but the second antenna has 11 Hz Doppler, which prevents channel phase synchronization. The Rx signal refers to the power of valve 0. The maximum input level of the receiver is −20 dBm (PER can exceed 10% for input levels above this value) [47,48].

The sensitivity of single or double reception using the 5.9 GHz DSRC module with a bandwidth of 10 MHz is shown in a scenario in Figure 7 with a lower packet error rate (PER) by approximately 12% in the 1000-byte physical service unit (PSDU) area for the input levels. 

Thus, the reception sensitivity is measured by an input signal directly on the antenna ports, and each touch or junction is blurred using pure Doppler. In addition, the second antenna uses 11 Hz Doppler, which prevents premature synchronization of channels [49]. The Rx signal refers to the signal strength by time 0. We can say that the maximum input level for the receiver is −20 dBm (PER can fluctuate, exceeding 10–15% in mobility conditions for all input levels). Regarding the characteristics and the transfer rate depending on the mobility of the devices, losses are expected, which ideally tend to zero, and in conditions of congestion and increased mobility, there is the possibility of interaction at distances of about 1000 m, as shown in Table 2.

For each derivation mapped using pure Doppler, we notice, in the case of the secondary Doppler antenna records, 11 Hz with a phase synchronization of the channels. The operability of the system in meteorological conditions reaching temperatures above +25 and +30 °C does not affect the sensitivity as it records noise of 1 dB even in conditions of increased mobility, as shown in Table 3.

**Figure 8:**
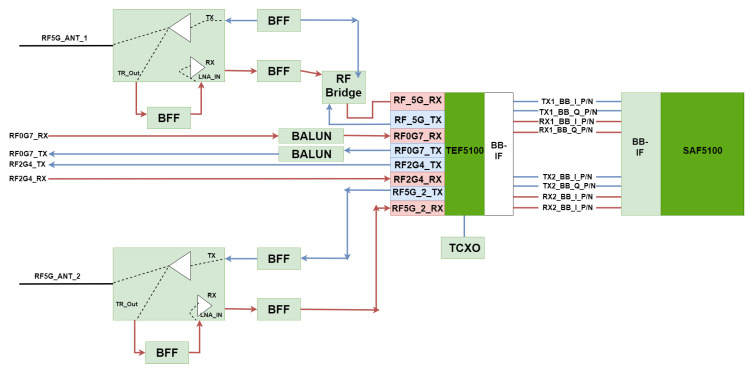
Front-End RF block diagram.

The mobility offered by PHY in the case of the IEEE 802.11p protocol is complete at the physical level of the layers with direct links to the PHY transceiver made by Cohda for cascading processing made in the main receiver. According to the RF and PHY interface, many radio solutions can be modeled, all based on the MK5 module, with implementation being possible in a single or dual environment. RF subsystems provide separate ports for 5 GHz antennas, without intercalating with the dedicated Rx or Tx outputs or pin sets of the 760 MHz and 2.4 GHz connections. Under the conditions of the duality of the configuration, the effective PHY mobility operates independently on each layer of the module, performing the operation on a different radio channel. This aspect can be analyzed by the configuration in Figure 8. In the case of 760 MHz and 2.4 GHz, RF circuits require external permission from the internal controller to switch the operational set. Therefore, the Cohda PHY mobility offers two antennas that can transmit and receive information on a frequency of 5.9 GHz in optimal performance conditions. In the case of the modes of operation on this frequency, the capacity of the system takes into account the following elements:

○ Single-channel mode (one or two antennas for different operations);
○ Dual-channel module (one antenna for each channel), two independent on IEEE 802.11 p and operable on different radio channels;
○ 10 MHz (DSRC) broadband channel mode;
○ Transmission via IEEE 802.11p Class C network mask (5 GHz band);
○ IEEE 802.11p improving the performance of the adjacent channel;
○ Cyclic transmission of the antenna delay (two operations in the 5.9 GHz band for each antenna);
○ Power control transmission (0.5 dB steps); and
○ Rapid changes in the way synchronized systems are communicated.

**Figure 9:**
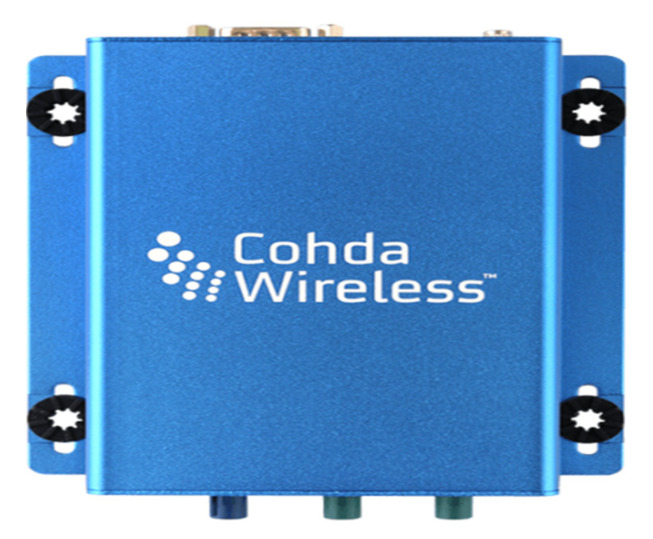
Communication module MK5 (OBU).

Regarding the communication mode, the MK5 platform shown in Figure 9 offers the possibility of using both the remote connection mode and the interface via the VGA port. The ability of the platform to operate and provide timely information is also due to U-Blox. Dedicated ports and pins for data transmission and the system are SAF5100 and TEF5100 IC. Out of a total of 62 pins found within the platform, approximately 10–12 can be customized according to the future requests of developing applications [50,51]. The safety elements are present in all cases, whether we are talking about the information storage or power supply part, with the latter being equipped with a voltage filter in case there is a faulty power supply regarding the car on which the system is installed. The presence of a DSP reception filter makes the information more accurate compared to other existing systems. The embedding of the system inside the vehicle in its current form is extremely easy due to its dimensions.

### 3.1. Analysis of the V2X System According to the Manufacturer

According to the previous specifications, the V2X system must adapt to the requirements imposed by the standard and the rigor of the legislation in force. In this direction, all the hardware elements of the device were analyzed in detail to expose it in scenarios that demonstrate the limitations it has in the case of vehicle applications. In the first stage of performance evaluation, indoor controlled scenarios were assessed in which measurements were made on a maximum distance of 45 m, with the transmission and reception part located on the outside of the buildings because in conditions of shielding or isolation, the GPS antennas do not provide stable communication and automatic synchronization is extremely difficult to establish. This can be considered a disadvantage in terms of communication between vehicles when traveling on a route that has either an underpass or a passage or even a parking lot of commercial space. Therefore, in conditions of extreme mobility, elements that do not meet the extreme requirements of users can be outlined and they can automatically be improved in future implementations. The evaluation of the V2X system performance will be performed over 10 mixed scenarios in which standard elements will be tested without increased mobility. 

### 3.2. Evaluation of V2X System Performance

The provision of two separate antenna ports for the 5.9 GHz band facilitates single-channel and dual-channel operation as well as two independent IEEE 802.11p radios. To improve radio performance, it is possible to transmit and receive diversity through two antennas. More precisely, the MAC layer running on the ARM processor of the SAF5100 allows a wide range of operating modes. We can say that this is like a single radio that shares the synchronized channel with other channels. The MAC address also includes measurements of other radio channels, such as channel statistics, the number of packets transmitted, lost, or waiting. This is extremely necessary for the processing and transmission of information [52].

We can say that the minimum transmission power is −10 dBm and the maximum transmission power is +22 dBm on the antenna port. This transmission power is controllable in steps of 0.5 dB each. Position detection and accuracy make the synchronization process crucial in the case of a V2X system. Therefore, the MK5 uses a GNSS receiver that includes GPS and Glonass support and Galileo to be able to replace it in conditions where the GNSS radio signal is weak, such as in urban areas with mixed relief and extreme high-rise buildings, to exclude phenomenon-faded calculation. The use of a gyroscope or data received from the CAN bus, such as speed, mileage, or directional angle, increases the degree of accuracy for the entire process analyzed. Therefore, the MK5’s GNSS receiver offers extremely accurate position and relativity upgrades at speeds of up to 10 corrections/s, with a horizontal accuracy of approximately 2 m and a precision synchronization equivalent to 25 ns. According to the previous chapters, the ports that benefit from the mixed-use background and in particular the serial port aim to connect the external GNSS receiver [53]. MKx runs a GPS (Daemon) server in the background that always has connections regardless of whether it is intermittent in the process of its transmission being connected without the data transmission being affected. We can say that this feature easily provides applications to access GPS data directly from the receiver, and from the command line for testing to optimize the operation of the GPS receiver [54].

### 3.3. Configuring V2X and V2V Architecture in Experimental Scenarios

In this section, we will highlight the key features of the implementation based on the elements discussed above. This includes analysis and visualization of the relevant information in the scenarios, interoperable and extensible implementation using network messages, and extraction and presentation of information on system traceability in different conditions and environments based only on the standard without improvements to deviate from it. 

Therefore, the architectural implementation of the system is based on a network interface controller, CAN modules, and the MKx platform, and for the data assimilation and connection maintenance part, a GNSS-GPS antenna. The transmission of information based on 802.11p is controlled by the ITS application on which the module is located and can have external access via an Ethernet or RS232 port, which is busy depending on the scenario presented, being useful for times when you want to troubleshoot unclear sequences or if problems are encountered in this process.

**Figure 10:**
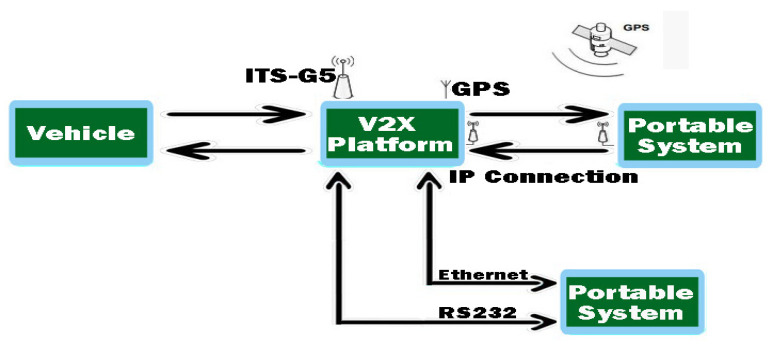
V2X communication diagram.

**Figure 11:**
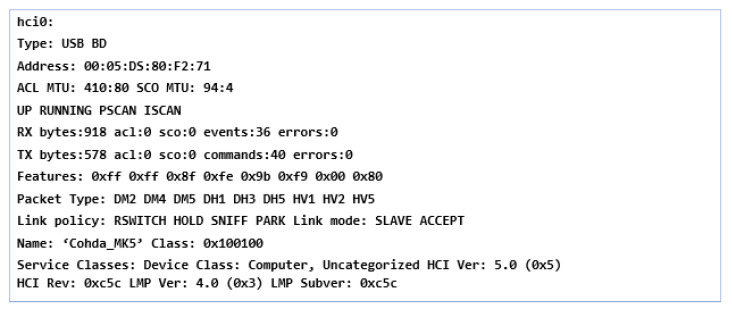
Initialization of the device and the host control interfaces.

As we can see from Figure 10, the whole concept and configuration of the system on which the scenarios are based that will analyze the capabilities and performance of the system in various traffic modes are presented. The configuration of the module and its synchronization with the whole system can be done using the host interface and by activating the accessible pins in/usr/etc/cohda/rc.inc (RC_LOCAL_PIN_DEF) or through fw_setenv_rc_local_pin “ID”, default 0000. The terminal loads the necessary Linux Kernel modules and activates btusb.ko, i2cusb.ko, i2cap.ko and also rftx-rxcomm.ko. The device is then initialized, and the host control interfaces are activated for equipment management and user recognition. To obtain the operating stage it has at that moment, the device can receive commands, such as *hciconfig-a*, having a similar result like this, through which we obtain information, as shown in Figure 11.

**Figure 12:**
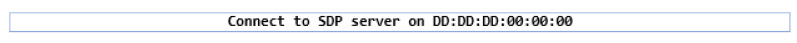
Connection to the SDP server.

This process confirms the information on the device name and the procedural use of authentication and initialization of the terminal but also the scanning of *hcitool* processes. Before setting up a connection, the standard and a simplified serial port profile are defined by configuring the SPP and replacing the RS-232 based on the RFCOMM profile, which performs a set of transport protocols to emulate the serial port. The typical point-to-point connection realizes the data flow in a flexible way and permanently supports the connection between the components of the MK5 module. Identification services have a unique 128-bit universal id (UUID), which is characterized by the example (SPP: 0 × 1101). The configuration of the utility and the programming of the SDP query evaluation interface for devices can be controlled via *sdptool* locally or via SSH, from another device, as shown in Figure 12. We obtain the connection confirmation in this system and manual activation is no longer required in this case.

### 3.4. Test Scenarios in an Indoor Controlled Environment

The stage preceding the external scenarios is performed in ideal test conditions and is perfectly controllable from all points of view. This element helps to configure the system before being exposed to the external environment to verify the dedicated communication over short distances but also the transition to the V2X test stage, which includes the control unit, external management system, and V2V communication.

**Figure 13:**
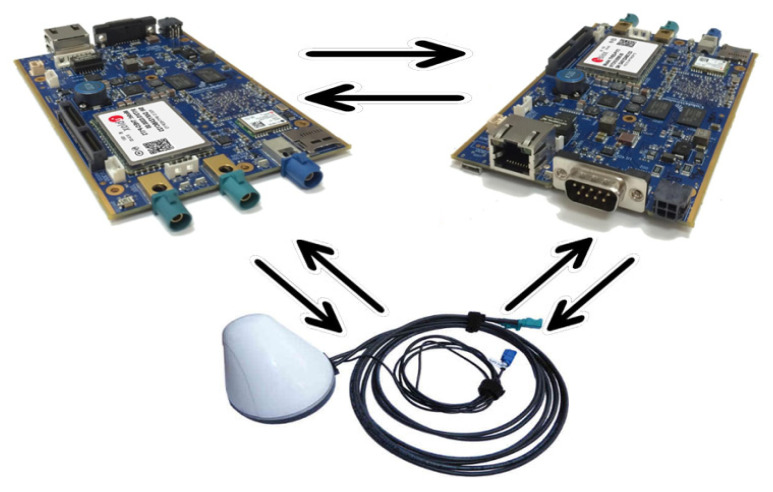
OBU 1 and OBU2—Antenna GPS—GNSS.

Therefore, these two components are integral boards that use and run Linux systems (Ubuntu 16.04 LTS), accepting the DSRC-V2X communication protocol, with the figure above illustrating the initial DSRC connection between OBUs and antennas, as shown in Figure 13. OBUs are supplied with 12V DC with a filter on each system to prevent voltage fluctuations that may exist in vehicles/networks. Their connection under laboratory testing conditions benefited from the configuration of a router as a server and IP addresses were assigned to each device as a unique identifier. The query and the physical connectivity between PC-OBU and RSU will be presented in stages depending on the stage of the simulations. In the case of the two OBUs and the internal simulation, it can be performed on the same sequence without distinction. The first step is to initialize the devices regarding the IP addresses that the system and the physical connection will assign. 

To make it easier to use devices in the future without the need for additional synchronizations and settings, the following initial device IP setup operations will be performed. The initialization of the devices is defined as standard, and they receive the IP address as 169.198.ABC.DEF and the last part of the six-digit IP address represents the hexadecimal value of the last four digits of the identification code of the serial number dedicated to the device. For example, a first initialization identifies a 76F9 conversion of a device to addresses, such as 118,249 terminations.

**Figure 14:**
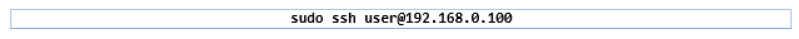
Command to connect to devices.

Therefore, the equipment needs in the first stage synchronization between the PC/laptop and DSRC equipment, which is in the same local network to be able to connect the whole system. Thus, after setting the IP addresses, in both the working system and the devices, having the same network segment, the first configuration commands can be entered in the terminal command. For this, we need a terminal or the installation of the Cohda operating system to be able to communicate. To connect to devices, we must authenticate its general use within the network by the command illustrated in Figure 14.

**Figure 15:**
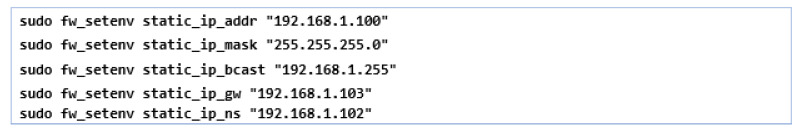
Identification and setting of the necessary IPs.

This sequence represents the main address from which we can authenticate the equipment, and we can initiate the following commands illustrated in Figure 15 in order to be able to identify and set the necessary IPs for the system.

**Figure 16:**
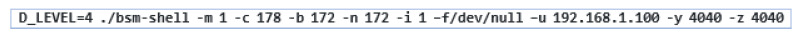
Identification of the directories associated with the module.

The sequence obtained represents the static IP addresses within the system, except for the device from which the queries are performed, which is identified by the address 192.168.1.101. Therefore, if the above steps have been performed, the following operation gives us the ability to remotely connect to the DSRC device at 192.168.1.100 followed by successive connections on terminations 102 and 103 to access the two OBUs in the network. In the communication, the computer becomes a control area, called superior, and the network created by the DSRC device becomes inverted, being controlled at every step by the instructions set by the user. The communication process of the computer/laptop under the control of the network can be done through bsm-shell. When there is information in the network from other DSRC devices, it is transmitted to the control area via the UDP protocol, and when the DSRC device receives messages via the computer/laptop UDP, it broadcasts them via the DSRC protocol. To maintain a stable connection without the need for constant synchronization, we must consider setting a function that executes the synchronization parameters each time the system is started without the need for user intervention. The location identification sequences are run: sudo scp bsm-shell user@192.168.1.100/home/duser, and the same process is performed for the other modules in the network that contain the/home/duser directory and the following command, as illustrated in Figure 16.

**Figure 17:**
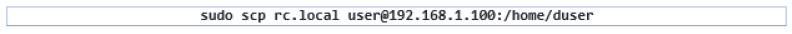
Creating a local rule file in the background.

This parameter synchronizes the instances and priorities of the system at the first start to streamline the process of receiving and transmitting the information. The bsm-shell is set to start automatically and then the PC/Laptop can indirectly use the DSRC protocol to be able to communicate with other DSRC devices by receiving and transmitting UDP packets. When the system is restarted, bsm-shell must be restarted in processes, either bsm-shell autostart elements are added or we create a local rc.local file that has the function of executable and works in the background, as shown in Figure 17.

The file with the location/home/duser is necessary to move to the/mnt/ubi directory, being found in cp/mnt. This will facilitate automatic running after each start. The configuration of the CAN port is necessary in terms of traffic speeds, although the automotive industry uses transfer rates between 500 and 125 kBit/s, which are common and are found in most systems. Alternatively, with other systems, maximum rates of up to 1 Mbit/s can be obtained. The CAN interfaces of the MKx modules can be set arbitrarily, thus supporting up to 1 Mbit/s for the configured file. In the case of Mk5, the files that contain information or act as a host and transmitter have the extension .conf, can0.conf, and cand1.conf with the location in, etc/int/, having a transfer capacity of 500 kBit/s.

**Figure 18:**
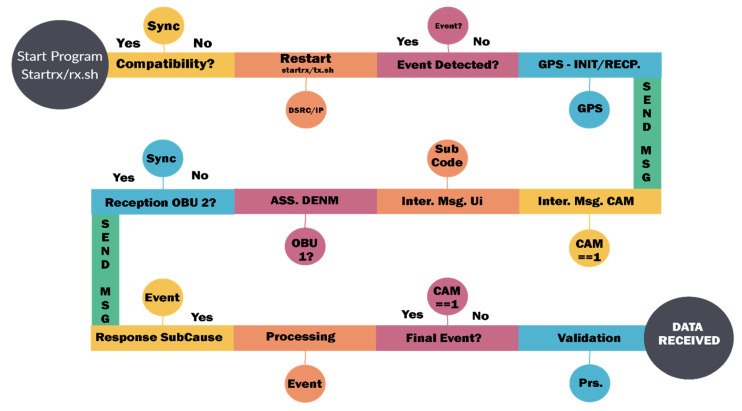
Data flow diagram between OBU1-OBU2.

According to the CAN specifications, the bit transfer time is divided into four segments: synchronization (fixed with a single amount), propagation, and buffer segment of phase 1 and phase 2. The length of each segment influences the resulting CAN bit rate, and the corresponding parameters can be set in/sys/devices/platform/FlexCAN.x/ or via the configuration file/opt/cohda/bin/rc.can, which is also recommended for system security and smooth running. There are certain necessary parameters for which the CAN rate can be calculated. Such a standardized configuration can achieve transfer rates of 1 Mbit/s with great weight, but there is a possibility to frame the value of 1.008786 Mbit/s as a tolerant one that can receive all packets on the CAN bus without data loss. Having all these elements properly managed can highlight the next steps we need to consider in this process. Outlining a data flow diagram becomes imperative in creating such a simulation model because certain functionalities of the system must be prioritized according to the requests we have to make in the scenarios. Therefore, according to Figure 18, the data flow diagram is outlined and is appropriate to the situations we will simulate. Using the Cohda Wireless Framework ETS Shell as a base provides a predefined structure that has added functionality to be extended later. The main program that contains being ets-shell.c and the one with the header have the extension *.h*, being an enumeration of the nearby stations in the network and that are listed after compiling it in the standard configuration. From this perspective, a flow chart is required on which the algorithm that will execute the data transmission and reception within the system will be applied. As we can see the data flow chart optimizes and prioritizes the information and functions needed to achieve communication, after setting the events and compiling, the basic program will initialize the CAN-GPS-UDP channels, then send test messages that must reach the other equipment in the system, and towards the end, an event must be outlined.

Each initialization function returns a descriptor file (integer variable), so the updated interfaces are accessed. If no information is available on the CAN interface, default values are loaded so as not to crash the entire system. The outline of the flow chart also aims to group the information and synthesize it to maintain a homogeneous flow without irrelevant data. You can see the functions for queries and those for limiting events and also the definition of descriptors that filter the information until it reaches a single place. The important information that we intend to extract and communicate in time within this system is the following: (Min-Max data packets, transfer rate, longitude, latitude, distance, signal for transmission/reception, noise for transmission/reception, and also the percentage of data lost if applicable). The prerequisite for the whole process is to synchronize the system modules and to have the whole circuit within the same network.

**Figure 19:**
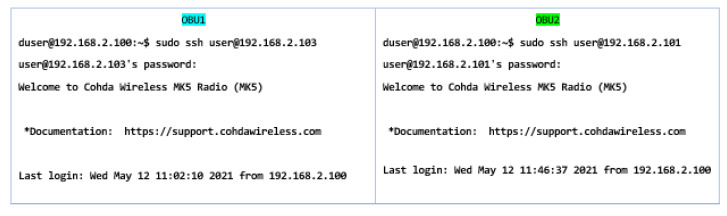
Authentication within the V2X platform.

After initialization, the system becomes fully functional and can be used in this way. The connection to the two OBUs on the central device that acts as a controller is made and subsequently, the desired scenarios are outlined. In this first stage, calibration tests and indoor scenarios are performed that are aimed at the final configuration of the system to expose it to the external environment and in mobility conditions. As we can see, duser is the main user who commands the entire system, and from it, by connecting to the encrypted serial port, ssh can authenticate each OBU that has been assigned an address in the system, as presented in Figure 19. Therefore, at this moment, the two devices are active and can run certain test scenarios to calibrate the internal components and also activate the GPS and GNSS functions or the ports dedicated to auxiliary connections or external memories.

**Figure 20:**
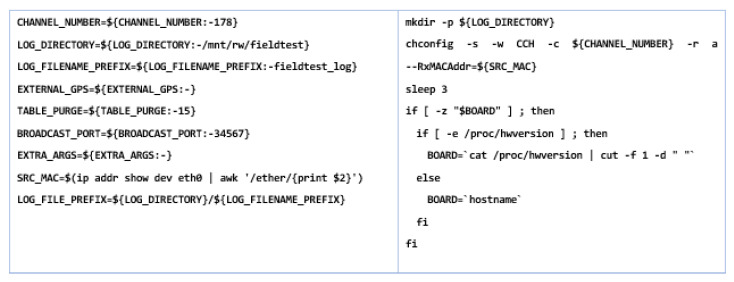
GPS and IP/MAC communication and activation procedure.

Therefore, after the complete initialization of the system, we must consider setting priorities on how the communication will be carried out and which of the two addresses will be the sender and which will be the receiver. The capacity of the modules presented in this chapter exposed the versatility they benefit from and the operation of any module both in the reception and in the transmission mode. According to the created file that is executed at system startup, several logs are made, and the writing directories are created. Then, the external GPS is activated and then the arguments for each port are set and the IP/ MAC addresses for each device are communicated through the general controller, also called the host, as shown in Figure 20.

**Figure 21:**
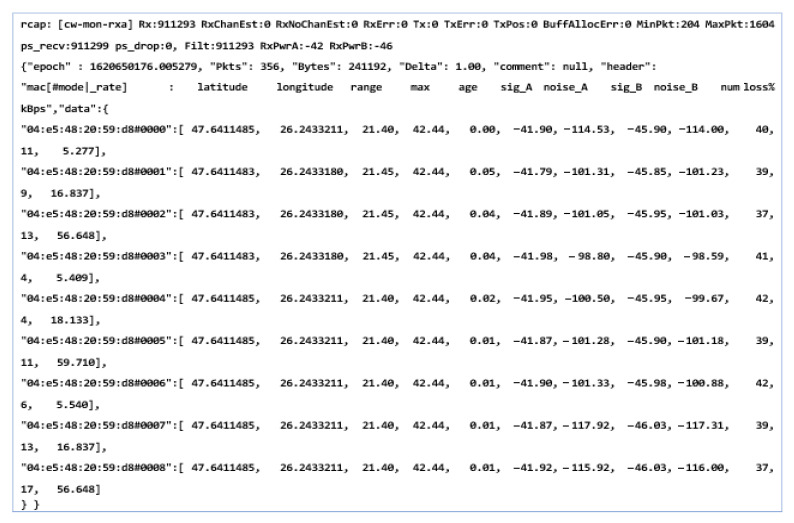
Initialization of the communication process.

This initialization and configuration process can take a few seconds to about 1 min depending on the coordinates that the system obtains and the data that it receives from the GPS antennas. We can say that the whole process achieved its goal and managed to display the information according to the coordinates from the satellites without positioning or location errors, and the transmission of data packets was successful without counting losses in a time of approximately 12 h. The result of the execution of the first set of queries is presented in Figure 21. It should be noted that we are talking about a static scenario without the execution of movements and without the dynamics of the components.

As can be seen, we obtain raw information on the entire process that takes place at the infrastructure level. This information has been established as a priority in testing a system with applicability in the automotive field and the field of road safety systems. This information is conclusive in identifying distances and locations and identifying the severity of the event by the strength of the transmitted signal and distance as well as noises or packets lost in the transmission and reception process. Another aspect that we have in mind is the one related to the limits that such a system could have, although the standard promises communication distances that exceed 800 m, without a clear specification of whether this is in conditions of high mobility, in a straight line, or with congested traffic. This is why all the properties and characteristics of the systems are taken into account to extract information over longer distances as well as in conditions and factors of extreme mobility. It is considered extremely important that a system based on the 802.11p standard can communicate smoothly and remotely up to 1–2 km regardless of the mode and dynamics of traffic. 

**Figure 22:**
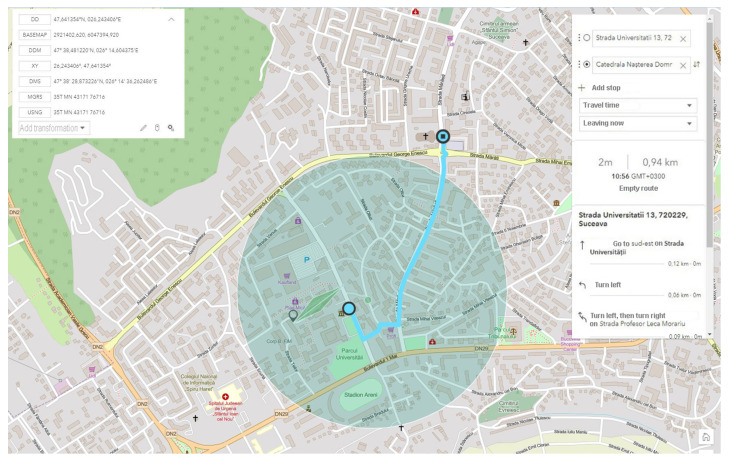
Complete scenario and simulated route.

Therefore, we can say that the indoor tests achieved their purpose and generated the expected results, outlining the general scenario in which the entire perimeter covered by this system is outlined. In Figure 22, we consider the exposure of the entire route and the starting points to test the system, with everything taking place gradually, starting from static parking scenarios and driving in a pedestrian area, with distances from close to close (5–10 m) between measuring intervals. Subsequently, these distances increase considerably, and the running speed is commensurate with the measuring intervals, as key points, also increasing exponentially (from 50 to 50 m and even from 100 to 100 m). In the final phase, moving wake scenarios for both OBUs in conditions of increased mobility, density, and distance between transmission and reception exceeding 1200 m in a straight line are investigated. According to the first query in which we analyzed the location and GPS coordinates, we have proof that the information is conclusive by comparing it with the map generated directly by Google’s CLOUD-API so that there is a permanent comparative model.

### 3.5. Test Scenarios in an Externally Controlled Environment

In shaping the external scenarios, controllable environments were prepared in terms of the flow of people and vehicles. This aspect outlined the first scenario in an outdoor environment located inside the university campus on an area of approximately 200–250 m of parking.

**Figure 23:**
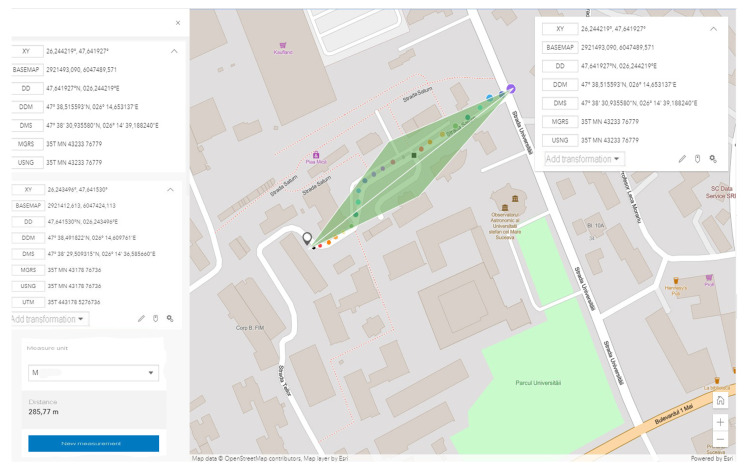
Controllable perimeter scenarios and covered area.

**Figure 24:**
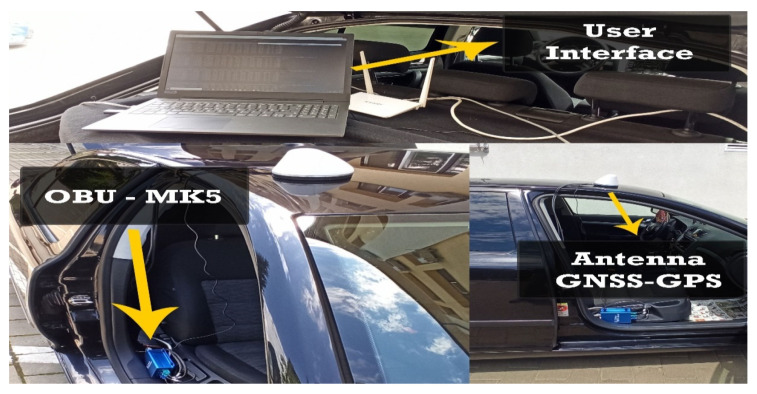
System configuration and integration in the passenger compartment (reception).

The starting point in shaping this test environment was ideal in terms of the fact that emission points can also be highlighted, as well as transmission-reception processes in motion. For optimal system testing, measurement and verification points have been set at time and distance intervals so that the degree of comparison of the identified location with that of the transmission can be identified. According to Figure 23, we can observe the starting point and the distance at which measurements are made under controllable conditions without obstacles or pedestrian or road density. The adaptability of the system and the integration in the passenger compartment of the car were achieved by managing each existing space and positioning it in the free spaces next to the support pillars of the car at the front. Therefore, the reception and interface part will be found on the vehicle and the transmission part will remain immobilized on campus throughout the tests on an indicator dedicated to identifying the parking lot, for better stability. All these elements can be seen in Figure 24, being positioned in highly visible areas that are easy to intercept. The fixed position established for the traffic sign is intended to represent a point in an intersection that could be exposed, such as a traffic light or an intelligent transport system. The transmitter for the entire duration of the tests transmits constant and large data packets that could contain information about the position of messages dedicated to traffic safety.

**Figure 25:**
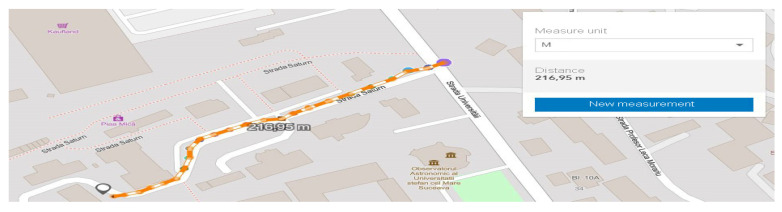
Route and total distance under controllable conditions.

**Figure 26:**
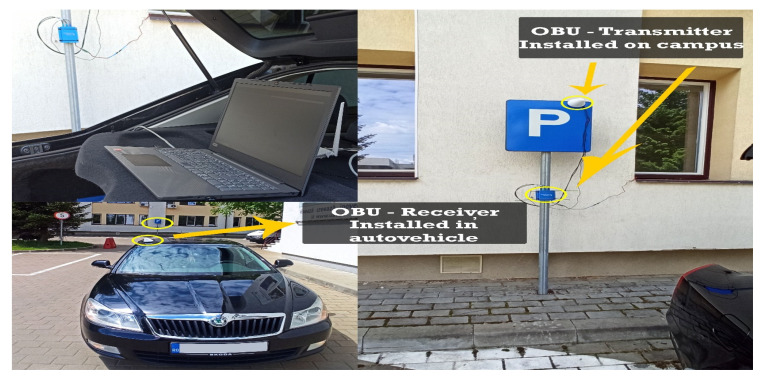
Transmission-reception communication system.

According to the standard, the conditions set out in these scenarios are ideal and throughout their duration, there should be no problems causing the connection to be lost over a distance of approximately 200–250 m, as shown in Figure 25. An audible warning or pop-up message on the user interface, according to Figure 26, highlights each interruption in the provision of packet data.

**Figure 27:**
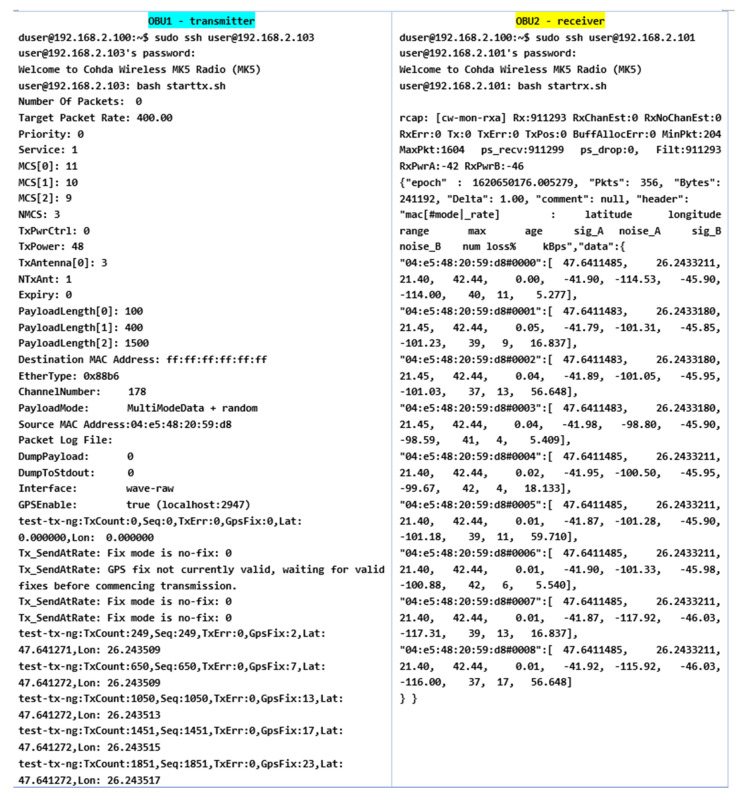
Synchronization algorithms between OBU 1 and OBU 2.

The information management and transmission application based on the MKx module runs the following main iterations to activate the communication interfaces and the transmission and reception based on CAM or DENM reception. Packet formation and their use as a common means of communication provide a protocol stack within the application in the form of an executable file that allows transmission and reception is the only way to manage this process because the source code of the central application is not open source. Using .ets-shell or .bash, we run the whole process and adapt the functionalities according to the main path/home/duser/mk5/stack/ets-shell to execute the main architecture (Mk5: ARM11, Mk4, or ARM Cortex-A9). According to the information transport process performed, special connections were initiated initially based on a common router on a frequency of 2.4 GHz, which depending on the distance and the test environment will switch to the frequency band of 5.8 GHz to perform the test process separately and completely autonomously from the client-server application. The main application manages the GPS information on the background of processes with direct writing in the file so as not to obstruct the data blocks received from other points. During this process, if no GPS signal is available, the gps-fake function is activated to transmit random GPS data (coordinates) so that later they can be synchronized when the signal returns, with the data being manipulated in the ~/x64/NNMEG file. .txt. The next step is when the system becomes active, feeds are waiting for a first test to identify a connection via a/ping/query in the main console. Therefore, we prioritize each MK5 module and set features, such as OBU1-MK5 (ID: 192.168.2.103) for transmitting and OBU2-MK5 (ID: 192.168.2.101) for receiving. Thus, the first order made to obtain the first transmission-reception process is executed according to the following rules presented in Figure 27.

**Figure 28:**
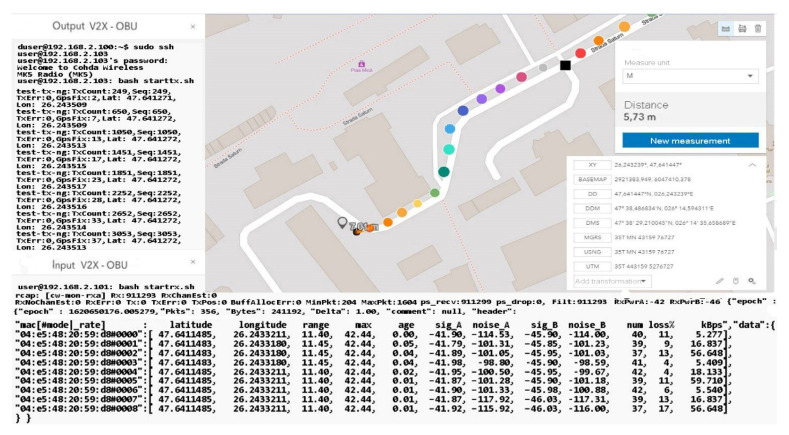
Simulated scenario at a distance of 7 m from the transmitter in a controlled environment.

**Figure 29:**
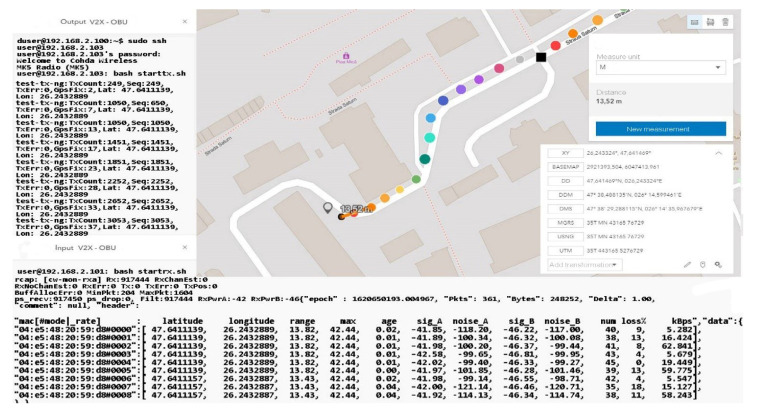
Simulated scenario at a distance of 13 m from the transmitter in a controlled environment.

As we can see, the initialization process was completed by setting the maximum data transfer target that must be transmitted, activating the GPS antenna, and initializing and synchronizing the MAC address for both transmission and reception. Therefore, this process of initialization and stabilization of the connection can take from 20–40 milliseconds to a maximum of 5 s depending on the area where the measurements are made and the GPS signal being affected by certain elements, such as tall buildings, congested areas, or antennas radio, in the vicinity of the area where the measurements take place. The first set of simulation scenarios is performed on the entire length of the parking lot from 5 to 5 m and from 10 to 10 m, with the measurement points being mandatory, however, in such conditions, changes can be observed in terms of the location and coordinates, being much more eloquent changes, as shown in Figure 28 and Figure 29.

The synchronization of the systems in short-distance conditions competes without problems. According to the inputs received at the reception, the highest threshold of lost packets is counted next to packets totaling over 56 kBps, about 17%, while in other cases, the losses are negligible.

In the mobility conditions, with the car in motion, we notice that the loss of data packets stabilizes and higher percentages appear in the case of much smaller packets than in the previous case. It is possible to observe the stability of the system and its flexibility under the given conditions. Even if the distances from the emission are reduced, we obtain the power of the transmitted and received signal, which is largely identical to that of the previous scenario. The noise identified for both antenna A and B is found in the parameters without compromising the stability of the communication.

**Figure 30:**
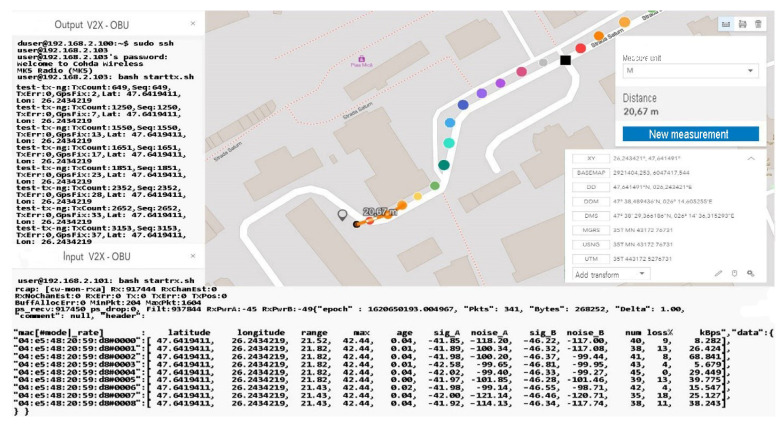
Simulated scenario at a distance of 20 m from the transmitter in a controlled environment.

**Figure 31:**
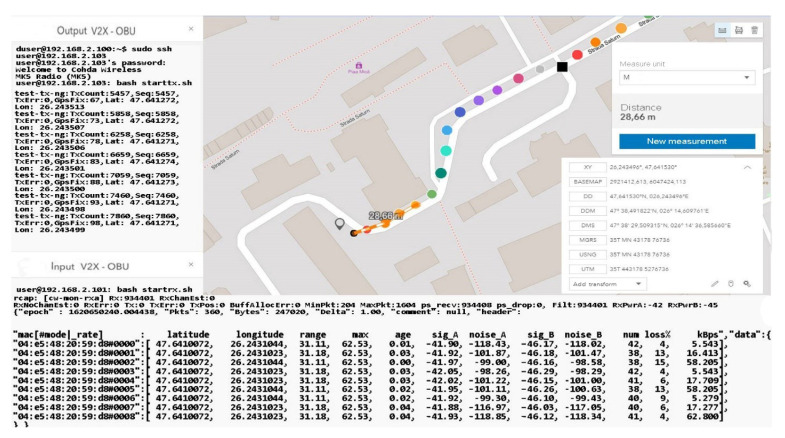
Simulated scenario at a distance of 30 m from the transmitter in a controlled environment.

The distance to the transmitter is constantly increasing without noticeable changes in data loss or disruptive factors that could damage the communication. Furthermore, the percentage of data lost for packets exceeding 60 kBps remains around 4–5%, and the amount of information is also in an ascending loop, as shown in Figure 30 and Figure 31. The noise signal ratio remains at the same linearity as in the case of the previous scenarios. Using the traffic and data, the generator periodically emits a signal every 100 ms, with systematized channel saturation being impossible. The performance of the signal-to-noise ratio according to the tests is identified by characteristics that highlight the accuracy and the degradation of the channel, and the imperfections of the devices are disturbed only in extreme conditions of extensive congestion. We can say that the IEEE 802.11p standard is based on three extremely important fields, such as the preamble, data field (GIS), and data flow, with the latter able to make the appropriate selection of the antenna and the correction of frequency synchronization compensations. The GIS field specifies the frame rate and length, encapsulating the MAC frame and the physical layer convergence procedure. Thus, the data field can have additional saturation, so its length can be a multiple of the bits encoded in an OFDM form.

Each pair of simulation parameters properly describes the PHY bitrate, which identifies the channel bandwidth and carrier frequency and also the types of antenna analysis. Here, we include the gain that describes the channel quality and connection of devices in the simulation process. The short-distance analysis confirms the degree of efficiency and stability of the standard in terms of data transmission from one vehicle to another that is on the same network and has common points of communication.

As we can see in Table 4, the classification of information and its extraction according to certain properties denotes reliability and an increased degree of stability. Some data are exposed with a negligible degree of error, at least in terms of distances, because the raw data containing the positioning coordinates are compared with the related ground data. Then, such a comparison establishes the error/noise and the automatic positioning, which can be different from the initial measurement by up to 7 m depending on the distance and the dynamics of the moment. The application of a post-processing and map matching algorithm points more to the notion of the degree of matching and the efficiency of the calculation performed.

Regarding noise factors, these occur when we focus on data analysis using GSM or OSM connections, as well as regression models to identify the factors that influence GPS observations in this context. The first set of scenarios performed measurements at distances of up to 30 m in ideal conditions from meter to meter to observe the behavior of the standard in the external environment. The presentation of the information in a concise manner aims to highlight only notable changes in the behavior and adaptability of the system to driving conditions. Therefore, the transfer rate remains constant, and the received packets do not encounter any losses that could cause the connection to be damaged.

In the case of these simulations that aimed to identify and limit a system in ideal parking conditions, all aspects of the rules and requirements of the standard as well as the legislation are taken into account. According to the results obtained, the measurements can be performed gradually depending on the distance, environment, dynamic, disturbances, and messages.

**Figure 32:**
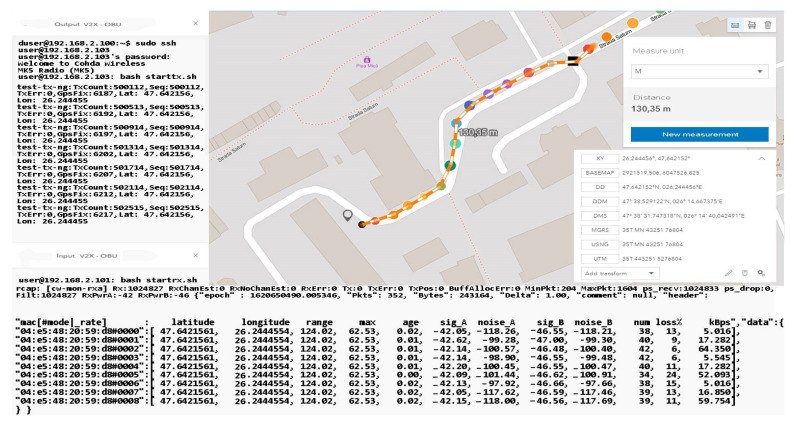
Simulated scenario at a distance of 130 m from the transmitter in a controlled environment.

As the distance increases and the measuring area becomes clean without buildings, the connection stabilizes and indicates a loss of 0 in the conditions of a packet of 65,350 kBps at a distance of 120 m, which subsequently tends to a loss of 11% in conditions of an equally large packet but at a distance of 130 m, as represented by Figure 32. The final measurement point under the conditions of this set of scenarios takes place at the boundary of the controlled area and the circulated area. The presentation of the obtained data can be found in Table 5, in which we can observe the regression in terms of losses according to the distance the vehicle is from the emitter. An exception is a scenario 75 m away. In this case, the vehicle reaches an area that has no obstacles, and the signal is not obstructed by buildings or other constructions. As we move in the direction of tests at considerable distances and with high mobility, we notice that the reliability and adaptability of the system depend on the conditions and factors that can degrade communication based on previous statements and the literature studied.

Under current conditions using two MK5—OBU devices, the measurements may also have erroneous predictability in certain scenarios or the Gaussian distribution exhibits a standard deviation of 0.5 m as exposed in previous chapters, which may create uncertainty in the measurement and identification of final coordinates. This can also be seen from measurements made when there are longer distances and repeated changes in the direction of travel. The distance between coordinates differs by up to 7 m in environments and conditions controlled with scenarios as static as possible or low to medium running. Given the use of an RSU-type road traffic unit that can operate in the dual-band and can maintain long-distance communication, the probability of much more eloquent results would be extremely high. We can say that the absolute average error varies with speed and shows an upward trend due to its relationship of direct proportionality between the running distance and speed, and given that with a value of 2 m compared to a speed of 60 km/h, the average absolute degree of error becomes extremely high. All these scenarios aim to approach the idea of a dedicated and ideal system to instrument an intersection and a DSRC communication flow. Testing the capabilities and limitations of a DSRC system leads to the development of new concepts and ways of coding in the process of structuring the standard and format of message transmission.

**Figure 33:**
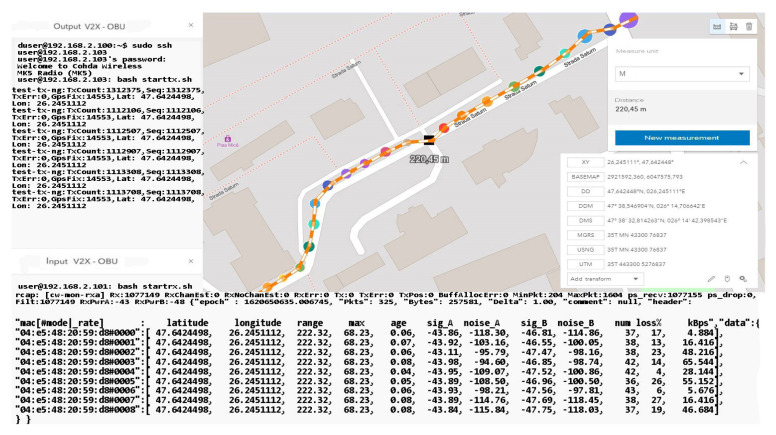
Simulated scenario at a distance of 220 m from the transmitter in a controlled environment.

The information quality depends strictly on the strength and accuracy of the available GPS signal, as well as on the filtering method that each OBU in the created network benefits from. In these scenarios, filtering is standard and OBU corrections applied by the system come from GPS antennas that reduce the estimated position uncertainty by about 2–3 for each longitude and latitude coordinate. This type of filtering is insufficient in the conditions presented in this paper, having different passages through different environments and complex measurements in high mobility scenarios, thus verifying the applicability of DSRC systems in V2R and V2X communication. In line with those mentioned above in the following scenarios, tall buildings are no longer found, but at the same time, the distance from the emission area significantly increases the signal, constantly deteriorating. Therefore, scenarios with average to low dynamics at a distance between 170 and 230 m will highlight the reduction of the delivery ratio of the data packets and the increase of the accuracy in the reception process, as shown in Figure 33. Another important aspect is that there is a possibility that data packets may be exposed to more severe collisions in congested areas or at intersections. Most testing for such equipment is performed in controlled environments without being exposed to actual practical cases where environmental conditions and unpredictability may pose extremely difficult difficulties for DSRC communication. Most of the experiments performed have a close outcome related to highways or poorly trafficked roads that can facilitate transfer rates or system stability. The current works do not consider a practical and applied modeling for the analysis of transmission path loss and testing in extreme conditions, as they disseminate the performance of packets in certain intersections based on a theoretical model of path search, using routes as a basis. If, in difficult conditions, certain buildings close to the route on which the measurements were made affected the visible line LOS, the communication remained reliable at more than 50 m from the emission area with effective radiated power (EIRP) of 30 dBm, which is lower than the limit imposed for these applications in Europe, namely 33 dBm. Under the controlled scenarios, the communication was sufficiently reliable and extremely stable at distances of 15–30 m and with an extremely low EIRP compared to the limit, ranging between 15 and 18 dBm.

According to Table 6, the signal decreases in some places, due to the increased measuring distance. This aspect does not jeopardize the process of data transmission and reception, but given that the network would have a fleet of cars, the data error will be much higher. We can say that following this scenario, we notice the limits to which DSRC communication begins to present the first inappropriate adaptations and deficiencies in the transport of information promptly. The measurements shall be performed without filtering and coding elements of the signals or messages in the network, with the information being raw and interpreted in a standard form. According to the literature and mentions made in other works, a large part of the highlighted problems can be corrected by filtering and information processing algorithms. We can say that the results obtained so far coincide with the theoretical works and the literature studied.

The creation of dependence is more than probable in the case of the received SNR, which is oscillating and presenting variations in its structure, automatically in the measurement process errors, or blurring of communication appears in certain measurement points. As the measurement operation advances in congested areas or is exposed to external factors, the error rate per packet increases compared to the received power, which has even become an exponential function in this process. It would be optimal in communications of this type to match the measurement data according to an exponential function, where deviation from the received power outlines a larger deviation than the reduced received power.

We can say that for a period of about 9 ms, the connection was blocked without realizing the exchange of factor information, which also caused the interruption. Later, it was able to receive GPS coordinates, and communication with the broadcast was restored, but for a short time in short pulses. The analysis of this behavior of the system shows that in extremely heavily trafficked urban areas with increased dynamics, the loss of connection and data transmission is affected. The data flow was interrupted about 300–350 m after the last display.

**Figure 34:**
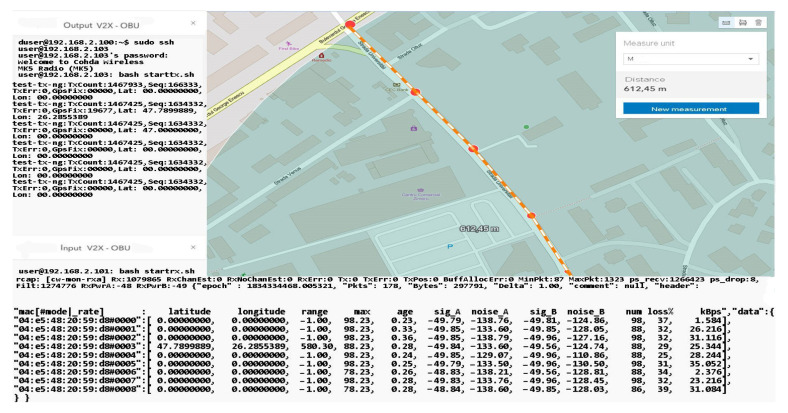
Simulated scenario at a distance of 612 m from the transmitter in an environment.

**Figure 35:**
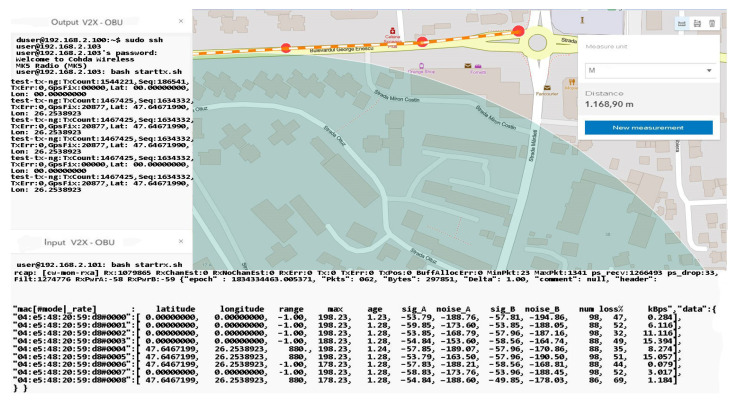
Simulated scenario at a distance of 880m from the transmitter in an environment.

This distance traveled by car was made on a part of the road with a level difference of 50–60 m and with an inclination of over 30° to the right. The moment of resumption of communication occurred at the maximum point calculated over a straight line distance of approximately 800 m with a total level difference of 75 m from the emission point, according to Figure 34 and Figure 35. Under these conditions, the measurements do not show a notable feature in view of the fact that the transfer rate is extremely low, and the number of packets received compared to those transmitted decreases exponentially. It should be mentioned that an extremely important aspect regarding these scenarios is that they are realized only with the help of two OBU-Mk5 modules without an RSU-type control center or another type of ITS system that could carry the information from node to node as close to the reception as possible. The data obtained in the last set of scenarios are presented in Table 7.

In the given conditions, without using a dynamic RSU-OBU type system in which the information would have been prioritized and transmitted through other nodes in the process through the network, we obtained these results. Most of the tests and measurements performed focused on complete systems, not by prioritizing messages from one OBU to another, with this aspect being a promising one using CAM or DENM messages. The results obtained are for relatively small packages according to the case code and sub-code specifications using the abbreviations in the nomenclature. The functionality of DSRC-V2X systems currently occupies an important place in the dynamics of the automotive industry and intelligent and autonomous transport. We must highlight the performance of the systems by optimizing them to the needs, not by vaguely understanding all the characteristics exposed. The current manuscript is at an early stage in terms of measurements, taking into account dynamic scenarios between vehicles and the information to be processed locally by each vehicle in portable navigation that will play the role of RSU in a dynamic form. The limitations of RF systems are imposed by the current development of infrastructure and the level of buildings that attenuate much of the signal. Technological advances in communications based on the 802.11p standard are blurred by these negative elements, such as traffic congestion, vehicle density, interference, tall buildings, and other disruptive networks. Even in the measurements performed, fluctuations of the connection stability were observed only by the level difference, with the fading of the signal being variable and dependent on several factors.

## 4. Discussion

Regarding the evaluations performed and the shaping of a measurement architecture for DSRC communication, we can expose several elements, among which the applications dedicated to DSRC lanes are those of active safety and services strictly related to the safety of passengers and pedestrians. The CAM-type messages of the WAVE standard, on areas with defined coverage, are extremely reliable and allow an increase in the level of road safety. They implement and use direct collision warning messages, lane changes or collisions at intersections, sudden braking, or uncontrolled movements by certain vehicles. These are active safety applications that can predict and prevent collisions in a cooperative way, unlike passive devices, such as seat belts and airbags, that minimize physical and material damage after an accident. According to the studies and to the results obtained in the extended simulations, the DSRC technology respects the requirements of the active safety applications, as it produces a latency of 0.2 microseconds. Another aspect is related to the devices that must detect vehicles, including image processors/cameras or LIDAR sensors capable of meeting the latency requirements. Many of the existing studies and applications propose cooperative systems in which the DSRC compositions are combined with shape recognition, subsequently generating an external map, and identifying the sender being processed by other sensor modules. There are also approaches to speed or the use of additional GPS devices to increase the accuracy of the data received.

The evaluations performed did not have the availability of a central data processing unit to constantly distribute the information. The times when the system had synchronization errors or problems establishing a connection were when the update period of the GPS antennas (tGPS) built into the Mk5 modules exceeded 200 ms. This conditioned the increase of latencies and automatically compromised the measurements and interrupted the connection for a few seconds. According to the data analysis and the initiated experiments, the main cause of the errors and failed synchronizations is the tGPS modules, highlighted by the distance calculation errors. Therefore, to minimize the influence of tGPS on the accuracy within the application, an algorithm is outlined to correct the warning and data transmission errors, which can reduce and increase these errors and increase the accuracy. The demonstrative part of the algorithm has not yet been updated and for technical-architectural reasons, it will not be possible to test or expose results in a short time without solid foundations through practical results.

**Figure 36:**
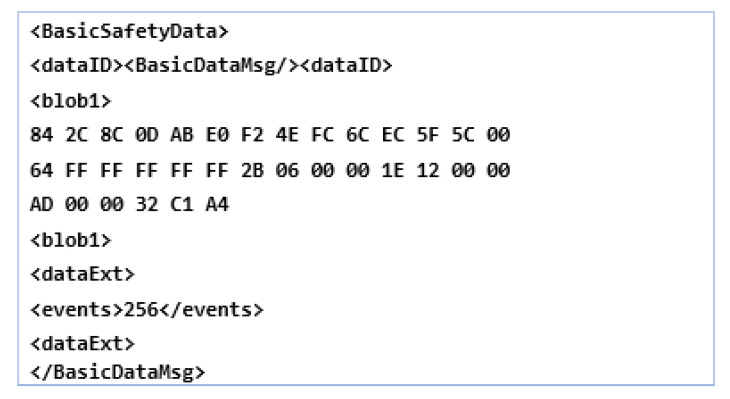
Basic security message structure.

Passing the algorithm over the standard information or obtaining the data through a single stationary vehicle query considerably reduces the error rate by finding the current position, current speed, elevation, or direction, thus encoding the data to build a BSM message through the ASN.1 standard. It does this without a recursion of iterations, already relying on the first set of data. The structure of a BSM test message transmitted to the control channel by the stationary vehicle (OBU1) has the above form. The main algorithm decodes the information via the GPS of the device on which it is run, calculating the distances, and monitoring the position and speed of the vehicle. The blob string also contains encoded information, as well as a message identifier in the form (0 × 75/116), with a timestamp (0× AB E0/4400 ms), machine latitude (0xF2 4E FC 6/C/26.247331, or longitude) 0 × E8 3C 6F 5 C/47.64312), altitude and speed, direction, and dimensions, such as length/width, as shown by the basic security message structure in Figure 36.

These messages are transmitted every 50 ms through the control channel to the DSRC. The presented approach outlines the elements of implementing a complete ITS system based on DSRC communications, but this approach is at the proposal level. The treatment and implementation of passive solutions to manage certain problems inside the passenger compartment will be presented in the next chapter.

We can say that the first stage provides sufficient viable prospects to obtain promising results in the implementation of infrastructure based on V2X-DSRC communications. Their ability to adapt to the context and to form ideal communication groups in vehicular or platoon applications can solve and stop a large part of the stated challenges. The inclusion of each car or street element in a network with intelligent systems and devices that communicate directly with vehicles can streamline and develop the area of vehicular communications.

## 5. Conclusions

The potential transmitted by the system and the architecture based on Mk5-OBU produced by Cohda Wireless can generate applications and field contributions through the ability to communicate in frequency ranges from 780 GHz to 5.9 GHz in the dual-band. We can say that given that the measurements were made in the first stage without using a complete OBU-RSU system, they are notable in terms of the fact that the coverage area is much larger than the specifications of the 802.11p standard. According to the scenarios, the maximum distance at which there was a connection between the two OBU-Mk5 modules exceeded about 900–1000 m in a straight line. According to the presented scenarios, the whole process was carried out in a congested area from a distance of 200 m from the transmitter, which was highlighted in this paper. If in the first stage the short distance measurements in a controlled environment highlight the reliability of the 802.11p standard, when the dynamics of the objects and the mobility of the vehicle were re-distorted, there were distortions or interruptions in the message transmission process. The studies carried out highlight the versatility of the standard in relation to the dynamics of the environment and its ability to adapt to the context in which it benefits from several routes and nodes in the network. Future directions are aimed at developing an RSU unit for the external environment, as well as a vehicle unit installed in the passenger compartment of cars in order to increase the quality and distances of communication. The elements highlighted in this article are based on the literature and present the usefulness of CAM-DENM messages in relation to the 802.11p standard, implementable in road safety systems and traffic congestion. In addition to all these aspects, a multitude of factors were identified that need to be taken into account, such as distance communication, duality, congestion, and interference. We can say that in addition to the previously mentioned directions, the creation of a mixed-hybrid DSRC-VLC network that would function as a V2X platform should be investigated. This could outline a new starting point in the development of applications dedicated to communication between infrastructure and vehicles or between pedestrian areas and local infrastructure, confirming the usefulness and capabilities of these standards in reducing road accidents or adverse traffic events by up to 80%.

## Figures and Tables

**Table 1:** Features, parameters, and specifications of the MK5 module.

Parameters	Specifications	
Frequency band	5.9 GHz, 760 MHz, 2.4 GHz	
Transmitted power	5.9 GHZ: −10 to +23 dBm	5.9 GHz: Class C
Reception sensitivity	5.9 GHz: −97 dBm	@ 3 Mbps
Alternative Antenna	5.9 GHz: CDD Transmission diversity MRC Diversity reception	
Band width	10 MHz, 20 MHz	
Transfer speeds	3 to 54 Mbps	
Power supply/Consumption	3.3 V și 5.0 V/4 W (maximum)	
Temperature range	−40 °C și + 85 °C	
Dimensions	40 (L) × 30 (W) × 4 (H) mm	
Standard	IEEE 802.11p—2010, ETSI ES 202 663, IEEE 1609.4—2010	

**Table 2:** Sensitivity felt by an MK5 module.

Number Channels ID	of Antenna MCS	Without Multipath [dBm] 1 Typical (Min)	Without Multipath [dBm] 2 Typical (Min)	Highway Nlos [dBm] 1 Typical (Min)	Without Multipath [dBm] 2 Typical (Min)
**11**	½ BPSK	−98 (−95)	−99 (−97)	−95 (−92)	−97 (−95)
**15**	¾ BPSK	−96 (−96)	−98 (−96)	−92 (−89)	−95 (−93)
**10**	½ QPSK	−97 (−95)	−97 (−95)	−88 (−85)	−92 (−90)
**14**	¾ QPSK	−93 (−90)	−95 (−93)	−86 (−83)	−89 (−87)
**9**	½ 16 QAM	−90 (−87)	−92 (−90)	−85 (−82)	−88 (−86)
**13**	¾ 16 QAM	−86 (−83)	−88 (−86)	−82 (−79)	−85 (−86)
**8**	⅔ 64 QAM	−82 (−79)	−84 (−82)	na	na
**12**	¾ 64 QAM	−80 (−77)	−83 (−81)	na	na

**Table 3:** Derivation-delay-Doppler processing table.

Derivation	Relative Power [dB]	Delay [ns]	Doppler Frequency [Hz]
0	0	0	0
1	−2	200	689
2	−5	433	−492
3	−7	700	886

**Table 4:** Simulation scenarios at a distance of 30 m from reception, data interpretation, losses, data transfer, signal, and noise.

Scenario/m	Packets/no	Distance/m	Signal [dB]	Noise [dBm]	Bytes/s	Stability/m	kBps	Loss
Scenario 7 m	356	11 m	−41.90/−45.00	−117	241,192	0.05	56.648	17%
Scenario 13 m	361	13 m	−42.00/−46.00	−120	248,252	0.04	62.841	18%
Scenario 20 m	341	21 m	−42.00/−46.81	−120.71	268,252	0.04	68.841	18%
Scenario 30 m	360	31 m	−42.02/−46.29	−118.34	247,020	0.04	62.800	15%

**Table 5:** Simulation scenarios at a distance of 130 m from reception, data interpretation, losses, data transfer, signal, and noise.

Scenario/m	Packets/no	Distance/m	Signal [dB]	Noise [dBm]	Bytes/s	Stability/m	kBps	Loss
Scenario 55 m	371	49 m	−42.65/−46.91	−119.2	244,024	1.62	61.212	20%
Scenario 75 m	371	77 m	−42.51/−47.29	−115.29	259,172	1.48	68.944	15%
Scenario 100 m	371	106 m	−42.27/−46.76	−117.07	259,172	1.43	58.195	20%
Scenario 130 m	352	127 m	−42.62/−46.55	−118.21	243,164	1.37	64.350	24%

**Table 6:** Simulation scenarios over a distance of more than 200 m from reception, data interpretation, loss, data transfer, signal, and noise.

Scenario/m	Packets/no	Distance/m	Signal [dB]	Noise [dBm]	Bytes/s	Stability/m	kBps	Loss
Scenario 175 m	349	175 m	−43.92/−47.33	−117.89	229,068	1.28	55.087	28%
Scenario 200 m	349	203 m	−45.97/−47.39	−119.89	229,068	1.19	65.067	28%
Scenario 220 m	325	222 m	−43.93/−47.75	−115.84	257,581	1.08	65.544	27%

**Table 7:** Simulation scenarios over a distance of over 650 m from reception, data interpretation, loss, data transfer, signal, and noise.

Scenario/m	Packets/no	Distance/m	Signal [dB]	Noise [dBm]	Bytes/s	Stability/m	kBps	Loss
Scenario 355 m	278	358 m	−58.85/−58.56	−128.16	297,581	0.18	45.151	36%
Scenario 521 m	278	528 m	−49.85/−49.96	−130.50	297,681	0.56	38.216	38%
Scenario 612 m	178	580 m	−49.85/−49.96	−130.50	297,791	0.36	35.051	39%
Scenario 1168 m	62	880 m	−49.85/−49.76	−189.07	297,851	0.88	15.349	69%

## Abbreviations

AODV	On-demand Distance Vector
ASN.1	Abstract Syntax Notation One
BRAN	Broad Band Radio Access
BSSID7	Basic service set identifiers
C-ITS	Cooperative Intelligent Transport Systems
CA	Cooperative Awareness
CAM	Cooperative Awareness Messages
CAN	Controller Area Network
CENELEC2	European Committee for Electrotechnical Standardization
CSMA	Carrier-Sense Multiple Access
DCC	Decentralized Congestion System
DEN	Decentralized Environment Network
DENM	Decentralized Environment Notification Messages
DNM	Default Mode Network
DSRC	Dedicated Short-Range Communications
ESO	European Level Organizations
ETSI	European Telecommunications Standards Institute
GBD	Global Burden of Disease
GDP	Gross Domestic Product
GNSS	Global Navigation Satellite System
GPS	Global Positioning System
ITS	Intelligent Transportation System
IVI	Initial Vehicle Information
LDM	Local Dynamic Mapping
LTE	Long-Term Evolution
OBU	Onboard Units
OFDM	Orthogonal Frequency-Division Multiplexing
OSI	Open Systems Interconnection
PDU	Packet Data Unit
PHY	Physical Layer
PSDU	Physical Service Unit
RFCOMM	Radio Frequency Communication
RSU	Road Side Units
SPP	Service Program Process
UDP	User Datagram Protocol
UMTS	Universal Mobile Telecommunications System
UUID	Universal ID
V2I	Vehicle-to-Infrastructure
V2V	Vehicle-to-Vehicle
V2X	Vehicle-to-Everything
VAG	Volkswagen AG
VANET	Vehicular Ad Hoc Networks
